# Supplementary material for: Imaging and SERS Study of the Au Nanoparticles Interaction with HPV and Carcinogenic Cervical Tissues
Source: Molecules. 2021 Jun 20;26(12):3758. doi: 10.3390/molecules26123758 (PMC8235590; doi:10.3390/molecules26123758)
Supplement: Supplementary file 1 [file molecules-26-03758-s001.zip › molecules-1242550-supplementary.pdf]

Supplementary information

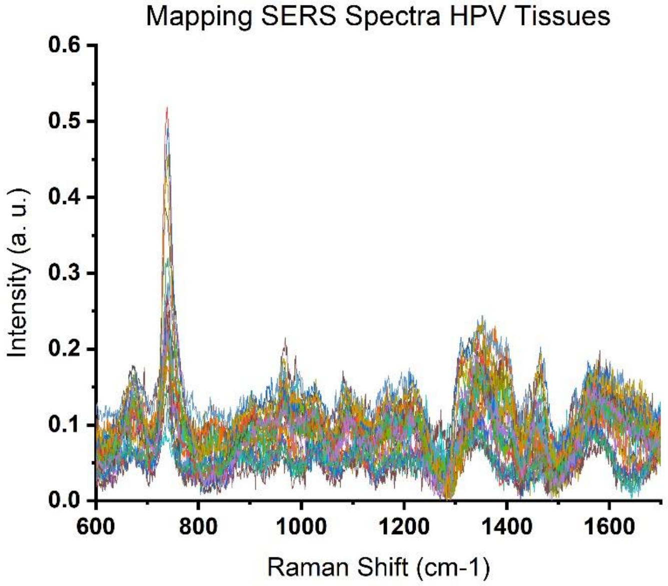

Figure S1 SERS spectra of HPV infected tissues mapping (n=30), analysed at a wavelength of 785 nm.

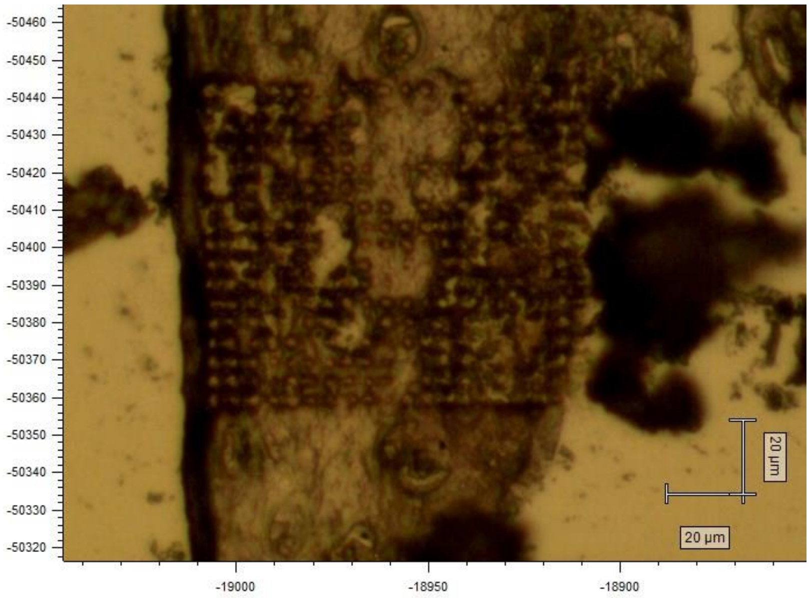

Figure S2 Micrography of an HPV infected tissue after the mapping raman análisis.

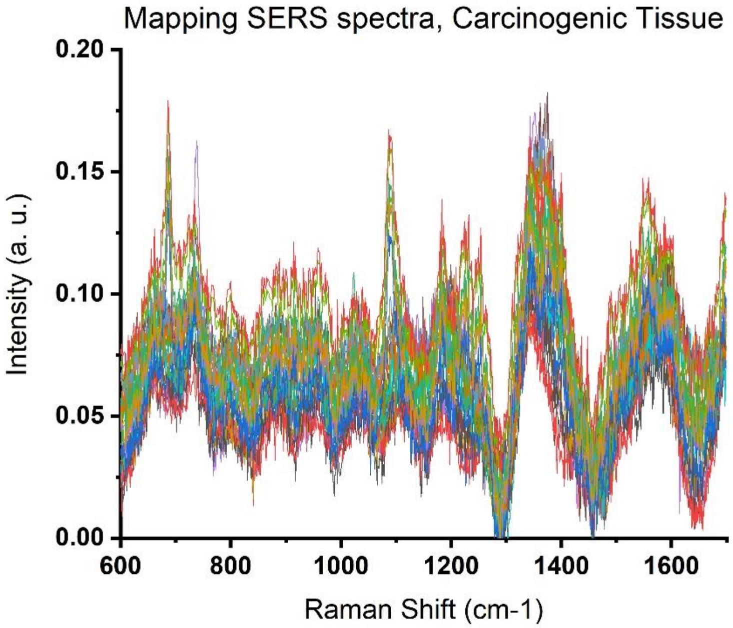

Figure S3 SERS spectra of Carcinogenic tissues mapping (n=30), analysed at a wavelength of 785 nm.

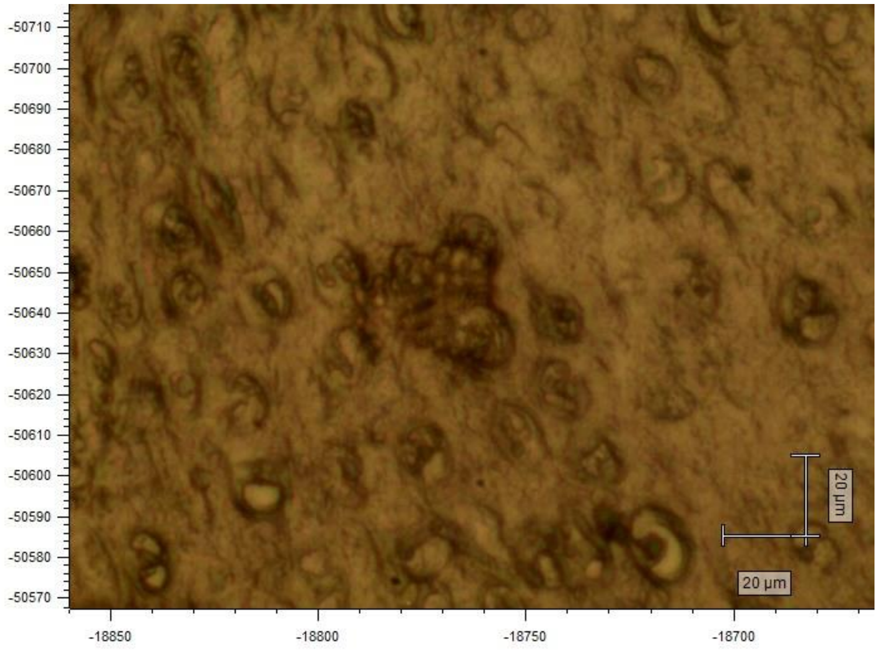

Figure S4 Micrography of a carcinogenic tissue after the mapping raman análisis.
